# Effectiveness of Immersive Virtual Reality on Orthopedic Surgical Skills and Knowledge Acquisition Among Senior Surgical Residents: A Randomized Clinical Trial

**DOI:** 10.1001/jamanetworkopen.2020.31217

**Published:** 2020-12-28

**Authors:** Ryan Lohre, Aaron J. Bois, J. W. Pollock, Peter Lapner, Katie McIlquham, George S. Athwal, Danny P. Goel

**Affiliations:** 1 Department of Orthopaedics, University of British Columbia, Vancouver, British Columbia, Canada; 2 Section of Orthopaedic Surgery, Department of Surgery, University of Calgary, Calgary, Alberta, Canada; 3 Division of Orthopaedic Surgery, Department of Surgery, University of Ottawa, Ottawa, Ontario, Canada; 4 Roth McFarlane Hand and Upper Limb Center, Western University Schulich School of Medicine and Dentistry, London, Ontario, Canada; 5 Canadian Shoulder Elbow Society, Canadian Orthopaedic Association, Westmount, Quebec, Canada

## Abstract

**Importance:**

Video learning prior to surgery is common practice for trainees and surgeons, and immersive virtual reality (IVR) simulators are of increasing interest for surgical training. The training effectiveness of IVR compared with video training in complex skill acquisition should be studied.

**Objectives:**

To evaluate whether IVR improves learning effectiveness for surgical trainees and to validate a VR rating scale through correlation to real-world performance.

**Design, Setting, and Participants:**

This block randomized, intervention-controlled clinical trial included senior (ie, postgraduate year 4 and 5) orthopedic surgery residents from multiple institutions in Canada during a single training course. An intention-to-treat analysis was performed. Data were collected from January 30 to February 1, 2020.

**Intervention:**

An IVR training platform providing a case-based module for reverse shoulder arthroplasty (RSA) for advanced rotator cuff tear arthropathy. Participants were permitted to repeat the module indefinitely.

**Main Outcomes and Measures:**

The primary outcome measure was a validated performance metric for both the intervention and control groups (Objective Structured Assessment of Technical Skills [OSATS]). Secondary measures included transfer of training (ToT), transfer effectiveness ratio (TER), and cost-effectiveness (CER) ratios of IVR training compared with control. Additional secondary measures included IVR performance metrics measured on a novel rating scale compared with real-world performance.

**Results:**

A total of 18 senior surgical residents participated; 9 (50%) were randomized to the IVR group and 9 (50%) to the control group. Participant demographic characteristics were not different for age (mean [SD] age: IVR group, 31.1 [2.8] years; control group, 31.0 [2.7] years), gender (IVR group, 8 [89%] men; control group, 6 [67%] men), surgical experience (mean [SD] experience with RSA: IVR group, 3.3 [0.9]; control group, 3.2 [0.4]), or prior simulator use (had experience: IVR group 6 [67%]; control group, 4 [44%]). The IVR group completed training 387% faster considering a single repetition (mean [SD] time for IVR group: 4.1 [2.5] minutes; mean [SD] time for control group: 16.1 [2.6] minutes; difference, 12.0 minutes; 95% CI, 8.8-14.0 minutes; *P* < .001). The IVR group had significantly better mean (SD) OSATS scores than the control group (15.9 [2.5] vs 9.4 [3.2]; difference, 6.9; 95% CI, 3.3-9.7; *P* < .001). The IVR group also demonstrated higher mean (SD) verbal questioning scores (4.1 [1.0] vs 2.2 [1.7]; difference, 1.9; 95% CI, 0.1-3.3; *P* = .03). The IVR score (ie, Precision Score) had a strong correlation to real-world OSATS scores (*r* = 0.74) and final implant position (*r* = 0.73). The ToT was 59.4%, based on the OSATS score. The TER was 0.79, and the system was 34 times more cost-effective than control, based on CER.

**Conclusions and Relevance:**

In this study, surgical training with IVR demonstrated superior learning efficiency, knowledge, and skill transfer. The TER of 0.79 substituted for 47.4 minutes of operating room time when IVR was used for 60 minutes.

**Trial Registration:**

ClinicalTrials.gov Identifier: NCT04404010

## Key Points

**Question:**

What is the quantifiable skill and knowledge transfer for surgical trainees using immersive virtual reality to learn both pathology recognition and complex procedural skills?

**Findings:**

In this randomized clinical trial of 18 senior orthopedic surgery residents, those trained using immersive virtual reality demonstrated significant improvements in knowledge and procedural metrics compared with a control group receiving technical video instruction. A transfer effectiveness ratio of 0.79 was demonstrated, indicating that immersive virtual reality substituted for 47.4 minutes of equivalent real operating room training.

**Meaning:**

These findings suggest that immersive virtual reality may play a significant role in the future of procedural training, supplementing and perhaps augmenting traditional teaching and effectively reducing early surgical learning curves.

## Teaser

This randomized clinical trial evaluates whether immersive virtual reality improves learning effectiveness among orthopedic surgery residents performing reverse shoulder arthroplasty.

## Introduction

Simulator use in procedural education of health care professionals is prevalent around the world. The use of simulators is supported by studies examining the comparison of, and transfer of skill training to, the real world.^1^ Combining skill transfer with training time provides an insight into the reduction of real-world training time.^2^ This has significant implications for cost-effectiveness and is particularly relevant considering the unknown long-term consequences of the coronavirus disease 2019 (COVID-19) pandemic on resident training disruption.^3,4^ Simulator analysis and validation has been used extensively in the aviation industry and military.^5^ However, it has been limited in procedural medical education.

Immersive virtual reality (IVR) offers a portable, multisensory, safe, and cost-effective experience. It has been previously validated during skills training in orthopedic surgery.^6,7^ However, further skill transfer must be studied to continue to demonstrate its value relative to real-life experiences. Given that traditional simulators rely on validated scoring metrics to determine effectiveness, the novelty of IVR should be validated by a similar metric.

Cost evaluation of resident surgical training is difficult to ascertain due to the difficulty in correctly identifying both direct and indirect costs. Variables to consider include regional variation and hospital structure, educational staff, facilities, clinical and operative duties, and salary. Direct educational costs per resident was US $134 803 in 2008.^8^ In academic year 2017-2018, there were 25 537 residents in surgical programs and 4760 in diagnostic radiology programs in the United States, which approximates to US $4.1 billion in direct training costs.^9^ Indirect costs are offset by Medicare in the United States, paying US $6.8 billion in 2010.^10^ These numbers do not account for opportunity costs of lost operating room (OR) time to all stakeholders, including surgeons. Considering the volume and potential cost of training, a simulator that provides a surrogate to real-world scenarios would be of considerable value to the medical community. With tens of thousands of trainees experiencing training disruptions as a result of COVID-19, educators must focus on providing consistent and efficient training strategies to supplement lack of real-world training opportunities.

Our hypothesis was that VR training would lead to improved technical skill compared with an instructional video. To determine this, scores on a validated surgical outcome metric, the Objective Structured Assessment of Technical Skills (OSATS) tool, between IVR and control groups were compared as our primary objective.^11^ Secondary objectives included comparisons of transfer of skill ratios with cost-effectiveness and validation of a novel IVR scoring metric, termed the Precision Score.

## Methods

The study was a randomized, intervention-controlled clinical trial of surgical residents to determine the effectiveness of IVR training in complex surgical skill acquisition. The trial protocol is available in Supplement 1. This study was approved by Ottawa Health Science Network Research Ethics Board, and all participants provided written informed consent. The study followed the Consolidated Standards of Reporting Trials (CONSORT) reporting guideline.

### Participants

**Figure 1.**
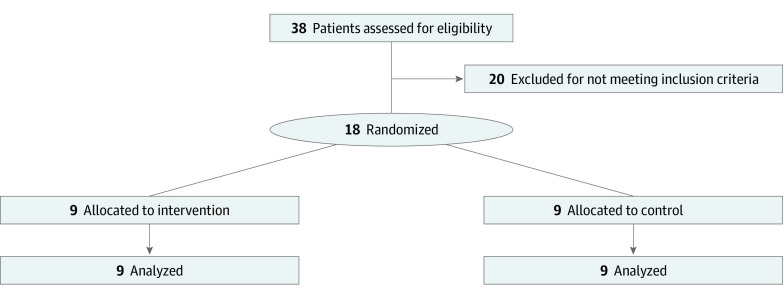
Study Flow Diagram

Senior orthopedic surgery residents (postgraduate year [PGY] 4 and 5) from 9 institutions who attended the 2020 Canadian Shoulder and Elbow Society (CSES) Annual Resident and Fellow Course were approached for study participation. Data were collected from January 30 to February 1, 2020. Three expert surgeons (A.J.B., J.W.P., and P.L.) who are members of the CSES were recruited to act as evaluators during the study. The study was conducted at the University of Ottawa Skills and Simulation Center. The study flow diagram appears in Figure 1.

### Randomization

Residents were block randomized using an internet-based, concealed computer-generated random allocation sequence. They were stratified by year of study into intervention (IVR training) or control (video training) groups. The intervention was not revealed to evaluators. Residents from both groups completed baseline Likert-scale demographic and confidence scale (CS) questionnaires (eAppendix 3 and eAppendix 4 in Supplement 2).^12^

### Training Intervention

Both groups received training on performing a reverse shoulder arthroplasty (RSA) for rotator cuff tear arthropathy. Cuff tear arthropathy predisposes the glenoid to a superior wear pattern, which necessitates implants with augments.^13^ The IVR group received training on the PrecisionOS platform version 3.0 (PrecisionOS Technology). The IVR module provides guided learning for key steps in the procedure and provides a composite Precision Score at the end of the module. The Precision Score is calculated based on several key parameters relevant to safe and successful implantation.^14^

The control group received training using a surgical video of RSA with augmented baseplate.^15^ Residents were provided with an iPad (Apple) and headphones and were instructed to replay the video as deemed necessary. Neither group was limited for repetition or learning duration. A third-party research member (K.M.) was present during each group training activity to mitigate bias of information. No member or affiliate of PrecisionOS was present during the entire course of the study. Both groups completed a written knowledge test and a repeated CS questionnaire following the training scenarios (eAppendix 3 and eAppendix 5 in Supplement 2).

### Cadaveric Preparation

**Figure 2.**
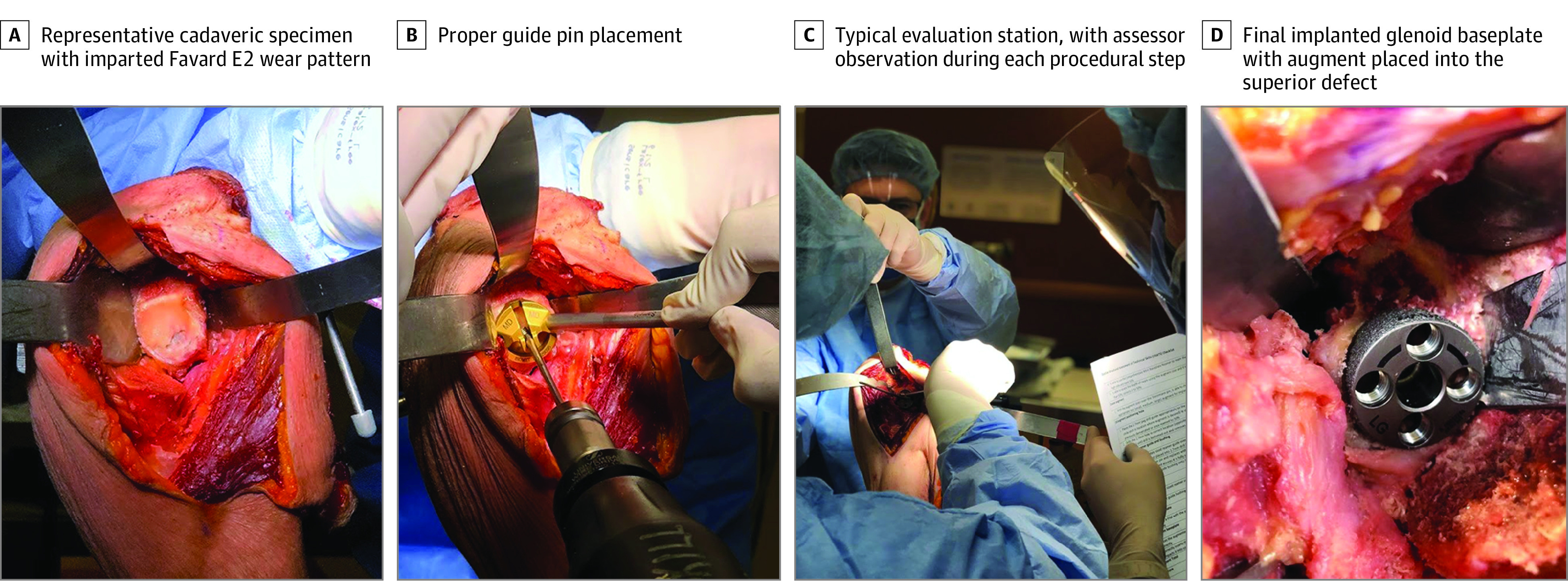
Representative Cadaveric Specimen D, Image uses different cadaveric specimen than other panels.

Fresh-frozen cadaveric specimens (scapula to hand) were prepared with a deltopectoral approach and superior glenoid wear pattern (Favard E2) (Figure 2).^13^ The superior defect was created using a standardized custom metal guide by the 3 expert surgeons (A.J.B., J.W.P., and P.L.). Cadaveric specimens were mounted on clamps. A third-party assistant as well as surgical device representatives provided by a medical device company were present to act as technicians and assist with surgical equipment. All assistants were instructed to limit interaction with study participants and not offer any technical advice.

Following the intervention, each participant was brought to the cadaveric lab to be assessed by masked evaluators for augmented glenoid implantation. Residents were asked verbal questions and were timed for their responses. Questions included identifying the location of the cadaveric wear pattern, predisposing condition to this pathology, visually identifying the wear pattern on a series of illustrations, describing the appropriate position of guide pin scapular exit, and describing the ideal ream depth for the wear pattern required for safe implantation (eAppendix 2 in Supplement 2). Residents were instructed on the role of assistants. The residents then performed an RSA using an RSA with augmented baseplate system (Zimmer Biomet). Residents were timed for task completion, starting from asking for or handling the first surgical instrument to final glenoid baseplate implantation. Prior to evaluating the participants’ performance, the masked assessors received training on the evaluation tools, including the OSATS and Global Ratings Scale (GRS) (eAppendix 1 and 2 in Supplement 2). Residents and evaluators were asked to provide subjective final implant parameters of version, inclination, rotation, and offset separately to avoid bias. Residents then completed final Likert-scale questionnaires consisting of learning activity assessment in parameters of realism, teaching capacity, and perceived longitudinal benefit as well as self-assigned GRS scores (eAppendix 6 and eAppendix 7 in Supplement 2).

### Outcomes

The primary outcome consisted of the OSATS score to determine whether there was any superiority using IVR training compared with control for learning decision-making and technical skills in complex RSA. Secondary outcomes include GRS, transfer of training (ToT), transfer effectiveness ratio (TER), and cost-effectiveness ratio (CER) scores of IVR training compared with control as well as validation of the Precision Score, a novel VR-based rating scale.

### Statistical Analysis

To achieve 80% statistical power (β = 0.2) for the primary outcome measure (OSATS), a 2-sided test at α = .05 revealed that a minimum of 6 participants was required for each group based on a conservative estimate of 25% difference in combined outcome measures of IVR training. Data were tested for normality prior to statistical analysis and analyzed by the intention-to-treat principle. We performed *t* tests for direct comparisons of means for normally distributed data for summative scores and Likert scales. We performed χ^2^ testing for normally distributed single Likert-type data. Pearson product correlation was used to determine similarity and correlation between ratings scales. We used Cronbach α to determine the reliability of Likert scales. Results were considered significant at a 2-tailed *P* < .05. Data were handled as a complete-case analysis, and all analysis was conducted in R version 3.0.1 (R Project for Statistical Computing).

## Results

### Participant Demographic Characteristics

A total of 18 study participants were randomized to control (9 [50%]) or intervention (9 [50%]) groups and did not significantly differ with respect to age (mean [SD] age, 31.0 [2.7] years vs 31.1 [2.8] years), gender (6 [67%] men vs 8 [89%] men), training level (PGY 4, 5 [56%] vs 4 [44%]), hand-dominance (right, 8 [89%] vs 7 [78%]), prior experience in shoulder arthroplasty, as measured on a Likert scale of 1 to 5, with 1 indicating less experience and 5 indicating more experience (mean [SD] experience with RSA, 3.2 [0.4] vs 3.3 [0.9]), experience in simulation (had experience, 4 [44%] vs 6 [67%]), experience with any VR products (had experience, 2 [22%] vs 5 [56%]), or experience with surgical videos (9 [100%] vs 9 [100%]) (Table). Prior to the intervention, the resident groups had no significant differences in written knowledge scores (IVR group, 11.2 [1.6]; control group, 10.2 [4.1]; difference, 1.0; 95% CI, −2.1 to 4.1).

**Table.** Demographic Characteristics of Included Study Participants

Characteristic	Resident, No. (%)	
	IVR trained (n = 9)	Video trained (n = 9)
Age, mean (SD), y	31.1 (2.8)	31.0 (2.7)
Gender		
Male	8 (89)	6 (67)
Female	1 (11)	3 (33)
Undisclosed	0	0
Postgraduate training level		
4	4 (44)	5 (56)
5	5 (56)	4 (44)
Hand dominance		
Left	2 (22)	1 (11)
Right	7 (78)	8 (89)
Any corrected vision		
Yes	5 (56)	1 (11)
No	4 (44)	8 (89)
Subjective experience with shoulder surgical approaches, mean (SD)^a^	3.8 (1.0)	3.6 (0.5)
Prior experience with RSA, mean (SD)^a^	3.3 (0.9)	3.2 (0.4)
Shoulder specific surgical courses attended, mean (SD)^b^	1.4 (0.7)	1.2 (0.4)
RSAs completed acting as primary surgeon, mean (SD), No.^c^	1.4 (0.7)	1.4 (0.7)
Prior use of any simulator in training		
Yes	6 (67)	4 (44)
No	3 (33)	5 (56)
Prior use of any VR products in our outside of training		
Yes	5 (56)	2 (22)
No	4 (44)	7 (78)
Prior use of VR products in surgical training		
Yes	3 (33)	0
No	6 (67)	9 (100)
Prior use of instructional videos in training		
Yes	9 (100)	9 (100)
No	0	0

Abbreviations: IVR, immersive virtual reality; RSA, reverse shoulder arthroplasty; VR, virtual reality.

^a^ Rated on a Likert scale (1-5), with 1 indicating less experience and 5 indicating more experience.

^b^ Rated on a Likert scale (1-3), with 1 indicating fewer courses and 3 indicating more courses.

^c^ Rated on a Likert scale (1-4), with 1 indicating fewer RSAs and 5 indicating more RSAs.

### Resident Experience

Compared with the video learning group, residents trained in IVR reported greater enjoyment of learning (mean [SD] control group score, 3.1 [1.0]; mean [SD] IVR group score, 4.4 [0.5]; difference, 1.3; 95% CI, 0.20 to 2.6; *P* = .01), believed it was easy to use (mean [SD] control group score, 3.6 [1.0]; mean [SD] IVR group score, 4.4 [0.7]; difference, 0.8; 95% CI, 0.003 to 1.3; *P* = .02), and provided a greater capacity for teaching (mean [SD] control group score, 3.2 [1.1]; mean [SD] IVR group score, 4.2 [0.4]; difference, 1.0; 95% CI, 0.3 to 2.2; *P* = .01). This included domains of anatomy teaching (mean [SD] control group score, 2.6 [1.4]; mean [SD] IVR group score, 3.7 [1.0]; difference, 1.1; 95% CI, 0.5 to 2.2; *P* = .002), and general surgical steps (mean [SD] control group score, 3.4 [1.2]; mean [SD] IVR group score, 4.3 [0.5]; difference, 0.9; 95% CI, 0.1 to 2.1; *P* = .009). The difference in the domain of implant-specific surgical steps was not statistically significant (mean [SD] control group score, 3.6 [1.2]; mean [SD] IVR group score, 3.9 [0.9]; difference, 0.3; 95% CI, −0.8 to 1.6; *P* = .67).

### Training Intervention

Based on a single repetition, the IVR-trained residents completed their training session 387% faster than those in the control group (mean [SD] time for IVR group: 4.1 [2.5] minutes; mean [SD] time for control group: 16.1 [2.6] minutes; difference, 12.0 minutes; 95% CI, 8.8-14.0 minutes; *P* < .001). When the total experience was factored, the IVR group repeated the module 2 to 3 times and were still 155% faster than tge control group (mean [SD] time for IVR group: 10.4 [5.0] minutes; mean [SD] time for control group: 16.1 [2.6] minutes; difference, 5.7 minutes; 95% CI, 1.6-10.3 minutes; *P* = .008). Mean (SD) total IVR training time was 10.4 (5.0) minutes vs 16.1 (2.6) minutes for total video training time. Residents completed 2 to 3 module repetitions during IVR training. There was a significant difference in module completion time between trials (difference, 6.7 minutes; 95% CI, 3.4-8.9 minutes; *P* < .001), with an mean (SD) reduction of 6.7 (3.1) minutes (range, 1.6-10.4 minutes; 95% CI, 4.5-8.8) between the first and second trial. The CS showed no significant difference between groups for baseline or posttraining confidence, with an internal consistency of 0.88. However, a greater number of residents who received IVR training (4 [44%]) had positive increases in their CS scores compared with participants in the video group (3 [33%]), and 1 resident (11%) trained with video actually had a lower CS scores; however, these differences were not statistically significant (*P* = .10).

### Cadaveric Procedure

Residents who received IVR training demonstrated significantly higher mean (SD) cumulative OSATS scores than the video group (15.9 [2.5] vs 9.4 [3.2]; difference, 6.9; 95% CI, 3.3-9.7; *P* < .001). Residents in the IVR group, compared with those in the control group, showed significantly higher mean (SD) scores on OSATS key domains of guide pin insertion (4.1 [0.8] vs 1.7 [1.2]; difference, 2.4; 95% CI, 1.1-3.4; *P* < .001), glenoid bone reaming (4.4 [1.0] vs 1.7 [0.7]; difference, 2.7; 95% CI, 2.2-3.5; *P* < .001), and augmented baseplate sizing (0.7 [0.4] vs 0.2 [0.4]; difference, 0.5; 95% CI, 0.003-0.99; *P* = .04). Consecutive errors were analyzed for each of the first 3 domains of guide pin insertion, glenoid reaming, and augmented baseplate sizing. Overall, 6 residents (67%) in the control group erroneously performed all 4 of the first key steps in guide pin placement compared with 0 in the IVR group. All control group participants (9 [100%]) erroneously performed the first 2 steps in bone reaming compared with 2 (22%) in the IVR group. Lastly, 7 residents (78%) in the control group did not at any point determine appropriate implant size during the intervention, while all of the IVR group performed sizing. Considering these parameters, the control group demonstrated 50% more critical errors in the early procedure than the IVR group (65% error rate vs 15% error rate; difference, 5.5; 95% CI, 3.9-7.0; *P* < .001). Residents in the IVR group completed the cadaveric procedure faster than those in the control group, although the difference was not statistically significant (mean [SD] time, 17.1 [5.7] minutes vs 25.3 [32.5] minutes; difference, 8.2; 95% CI. −39.4 to 21.0; *P* = .13). GRS total and individual domain scores were not significantly different between groups. During the cadaveric procedure, the residents trained in IVR demonstrated significantly higher mean (SD) verbal questioning scores than those in the control group (4.1 [1.0] vs 2.2 [1.7]; difference, 1.9; 95% CI, 0.10-3.3; *P* = .03).

**Figure 3.**
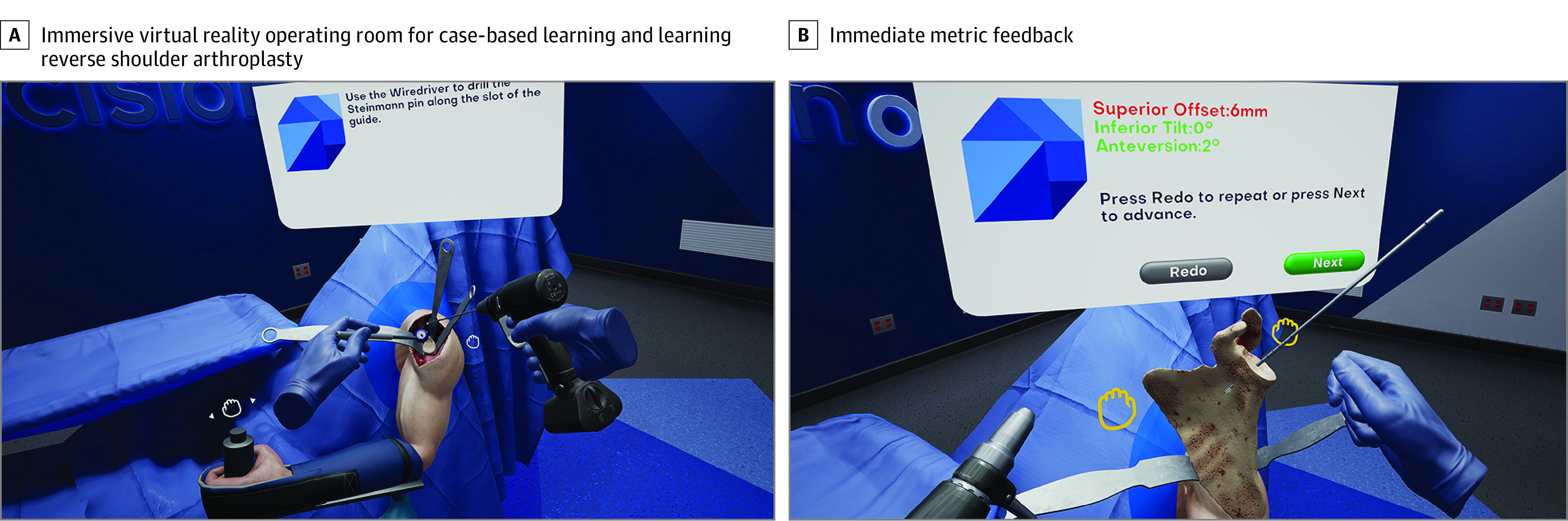
Immersive Virtual Reality Operating Room and Metric Feedback

Residents receiving IVR training were measured using a proprietary Precision Score, computed from composite data set of parameters in the virtual patient environment (maximum score is 1.0). The mean (SD) Precision Score in the IVR group was 0.75 (0.13) (range, 0.52 to 0.92). There was no significant change between Precision Score between module completions. The Precision Score revealed a strong correlation (*r* = 0.74) to OSATS scores, had good internal consistency (0.82), and correlated with GRS scores (*r* = 0.32) and completion time (*r* = 0.43). The Precision Score was observed to correlate with implant orientation parameters provided by expert surgeons (*r* = 0.73) for the final construct. Figure 3 depicts a representative Precision Score and example of the virtual training environment.

### Training Validity

**Figure 4.**
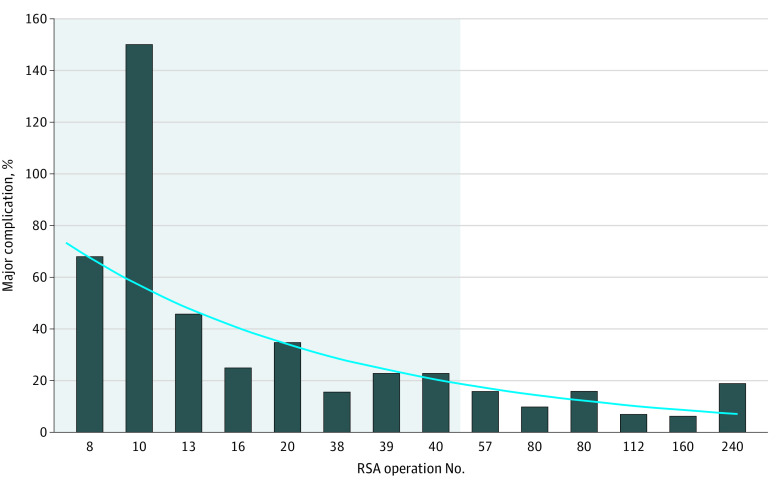
Fourteen Representative Studies of Early Learning Curves and Composite Major Complication Rates for Performing Reverse Shoulder Arthroplasty A total of 14 studies^13,16,17,18,19,20,21,22,23,24,25,26,27,28^ were used. A fitted curve (*r*^2^ = 0.80) allows for determination of early learning curve reduction based on transfer of training ratios. In 1 study,^26^ multiple complications occurred in operative participants, resulting in a complication rate of greater than 100%. Our transfer of training of 32.5% to 59.4% illustrates that immersive virtual reality training reduces early learning curves by 13 to 51 cases, represented by the gray rectangle and solid vertical lines.

The ToT, TER, and CER ratios were calculated for IVR training and compared with control training. The ToT ratio was 59.4% based on the cumulative OSATS score. The TER ratio was calculated as 0.79 when comparing performance of IVR training with control training. Figure 4 demonstrates the effects of ToT on early learning curves.^13,16,17,18,19,20,21,22,23,24,25,26,27,28^ The cost incurred for IVR training was considered a function of biweekly use on a US $4800.00 per year cost, equaling US $46.15/session. The cost of traditional training was considered the cost incurred to an individual for course registration and travel and was approximated as US $2000.00. Based on these estimates, the CER was 34.1. This does not account for opportunity cost in missed wages and is presented as cost incurred to residents.

## Discussion

By means of a randomized, intervention-controlled trial, IVR was demonstrated to be superior to technical video training for acquisition of procedural knowledge as well as pathology recognition and decision-making. The IVR-trained group had significantly improved OSATS scores as well as higher verbal questioning scores following a single training session. OSATS is a reliable means of determining training effectiveness across surgical disciplines.^11^ OSATS is a composite of key actionable steps during evaluation, all of which convey both procedural knowledge and technical ability. The control group missed a mean of 67% of the key steps in guide pin positioning and glenoid reaming, and only 2 (22%) decided to choose an appropriately sized implantable component. The IVR-trained group significantly outperformed the control group in these 3 key areas. Considering technical factors in the ultimate position of the glenoid component during RSA, initial guide pin orientation, amount of resected bone, and size of components are all contributable risk factors of early, catastrophic implant failure.^29^ The control group significantly underperforming in these areas demonstrates the importance of proficiency training in safe, simulated environments to prevent patient harm. A number of participants in the control group rapidly completed the glenoid implantation task, albeit incorrectly due to missing many key surgical steps, resulting in a nonsignificant difference in cadaveric implantation time. This highlights the importance of considering performance ratios rather than simple metrics alone.

Simulator research emphasizes ratios, including transfer of training experience to real scenarios. Using ToT and TER ratios, we can determine whether the simulator accomplishes the training task for which it was developed.^2^ This method of transfer validity has been used in medical education, surgical education, and in other industries, including flight and military training.^2,30,31^ Our study used an experimental-vs-control-group method of ToT and TER determination, considered the most appropriate for determination of validity.^32^

Our study demonstrated a ToT of 59.4% based on OSATS scores. ToT informs potential reduction in early learning curves by training with a simulator.^33^ Given that outcomes in RSA are directly related to surgeon experience,^33^ development of simulators that provide enjoyable learning with tangible skill or knowledge improvements related to training material is crucial for patient care. Based on the available evidence for early learning curves in RSA from multiple, experienced surgical groups,^13,16,17,18,19,20,21,22,23,24,25,26^ the ToT achieved using the IVR simulator would account for performing 51 RSA procedures. Considering the scalability of the platform, this ToT value may also be higher, given that training can occur on cases with varying complexity.

The TER accounts for time spent in the simulator compared with training time in the control environment, relative to real-world procedural time to reach task completion. The TER additionally provides information on potentially reduced training times by using a simulator. A TER of 1.0 indicates simulator-based training is equivalent to real-world training. Interpreting our TER of 0.79 reveals that 1 hour of IVR training is equivalent to 48 minutes of real-world training time. The reported mean flight simulator TER is 0.33,^31^ including those that are used for licensing. One of the most prominently studied VR simulators is the Minimally Invasive Surgical Trainer–VR laparoscopic trainer, which has previously shown a TER of 0.42.^30^ A direct comparison between simulators in varying fields is impossible because of system, task, and user variables. Consideration of real-world training reductions through simulation is important, and these other values illustrate successful implementation in other high-performance fields, even with less time-saving features.

From a cost perspective, 1 minute of OR time costs US $37.^34^ Considering the reduction in on-the-job learning time provided by the IVR, this has the potential to greatly affect procedural training time and costs. Safely reducing learning curves in a virtual environment reduces complication costs. In our study, a single episode of IVR simulation trained the residents to more correctly perform the critical steps of the procedure, identify surgical pathology and reconstruct a contextual surgical problem. Creating a pathological scenario in study cadavers represents high-level learning beyond simple task repetition and has not been previously shown. Based on these findings, we have shown a CER based on improved training time provided by the TER, relative to video training, and the cost of a single training course as a surrogate of traditional training methods. With these assumptions, the IVR training is at minimum 34.1 times more cost-effective than our control. From a residency program perspective, assuming it would send a mean of 20 trainees to a course that costs US $2000 course, the CER to the program would increase to 685, considering the cost of a single IVR headset used biweekly by each trainee. If we consider attending multiple courses or incorporating the per-minute OR cost, the cost-effectiveness further increases. When tens of thousands of trainees return to standard training, measuring and providing skills outside the OR will be essential to mitigate the costs associated with remediation. Doing so in a cost-efficient manner compared with traditional training and courses is a novel perspective.

Validated scoring metrics are available for open and minimally invasive procedures. These include the OSATS, GRS, and Arthroscopic Surgical Skill Evaluation Tool. These have previously been validated for skill acquisition and demonstration in real-world scenarios.^35^ To date, there is no objective rating scale for IVR given its novelty. The Precision Score was developed to determine training module outcome in the virtual world. A benefit of IVR is the ability to provide immediate metrics and feedback to users. The Precision Score is an adaptable score that incorporates time to task completion with evidence-based parameters of achievement. To our knowledge, our study is the first of its kind to validate the use of an IVR rating scale. The Precision Score measured strong correlation coefficients and internal consistency compared with OSATS performance. Important for procedural applications, the Precision Score also strongly correlated with both objective and subjective real-world improvements in implantation parameters. This adaptable score must be further used and validated to become the standard of outcome measurement with the increasing use of IVR technology.

### Limitations

This study has limitations, including small recruitment numbers based on volunteer convenience sampling. The confidence scale used may be too granular for single training sessions given task complexity. A perceived limitation is whether this technology applies in a longitudinal manner. We feel that this is not a limitation because IVR provides just in time education that is convenient and portable. We used cadaveric specimens rather than a real operative scenario; however, this has been demonstrated as an appropriate substitute.^1^ Furthermore, most studies do not attempt to create a pathologic situation in the cadaver to simulate the real life context. This study attempted to capture all relevant variables to simulate a situational environment a surgeon may face in this specialty. Consistency of scoring between evaluators could be improved with intervention recording and reassessment by multiple evaluators. We have additionally compared IVR training with technical video training alone and not with mixed media methods, which are commonly used by learners.

## Conclusions

In this study, learning complex procedural skills and critical steps in IVR was found to be superior to technical video training. The IVR training platform provided improved knowledge and pathology recognition to senior surgical residents in a single session. IVR training demonstrated significantly fewer errors and no egregious or critical mistakes. Validated, objective measures of training effectiveness demonstrated reduction in both theoretical early learning curves and real-world training time with IVR use. The TER value for IVR was more significant than prominent surgical simulators previously examined. The newly developed Precision Score, an IVR scoring metric, correlated with both technical skill in the real-world task and final product quality, thus simultaneously providing and validating a novel assessment tool for the virtual simulation environment. Further research into the effects of longitudinal IVR learning must be undertaken.

## Notes

Supplement 1. **Trial Protocol**

Supplement 2. **eAppendix 1. **Global Ratings Scale Document **eAppendix 2. **OSATS Checklist Document **eAppendix 3. **Confidence Scale Document **eAppendix 4. **Demographic Survey **eAppendix 5. **Knowledge Test Document **eAppendix 6. **Postactivity Control Group Questionnaire **eAppendix 7. **Postactivity Experimental Group (IVR) Questionnaire

Supplement 3. **Data Sharing Statement**
