# Supplementary material for: Effectiveness of Immersive Virtual Reality on Orthopedic Surgical Skills and Knowledge Acquisition Among Senior Surgical Residents: A Randomized Clinical Trial
Source: JAMA Netw Open. 2020 Dec 28;3(12):e2031217. doi: 10.1001/jamanetworkopen.2020.31217 (PMC7770558; doi:10.1001/jamanetworkopen.2020.31217)
Supplement: Supplement 1. — Trial Protocol [file jamanetwopen-e2031217-s001.pdf]

Transfer Effectiveness of Surgical Education with Immersive Virtual Reality: A Randomized Controlled Trial

Principle Investigator: Danny P. Goel, MD
Assistant Professor Department of Orthopaedics
University of British Columbia
Burnaby, BC, Canada

Author: Ryan Lohre, MD
Department of Orthopaedics
University of British Columbia
Vancouver, BC, Canada

Version: 1.0

Abbreviations:
Immersive virtual reality (iVR)
Statistical analysis plan (SAP)
Post-graduate year (PGY)
Reverse shoulder arthroplasty (RSA)
Head mounted display (HMD)

Table of Contents:
1. Introduction
2. Study Design
    a. 2.1 Sample size calculation
3. Aims and objectives
4. Outcomes
    a. 4.1 Primary outcome
    b. 4.2 Secondary outcomes
5. Populations and subgroups to be analyzed
    a. 5.1 Populations
    b. 5.2 Subgroups
6. Analyses
    a. 6.1 Primary outcomes
    b. 6.2 Secondary outcomes
7. Missing data

1. Introduction

The aim of this study is to characterize the role of immersive virtual reality (iVR) training in knowledge and technical skill acquisition in orthopaedic surgery through a multi-institution, single session educational randomized controlled trial of senior residents.

This statistical analysis plan (SAP) is provided for clarification of primary and secondary outcomes and analyses plus data management.

2. Study Design

This study is an educational, intervention-control, randomized trial incorporating senior orthopaedic surgery residents and consultant fellowship trained shoulder surgeons. Recruitment will be from attendance of the (removed/blinded) annual resident course and meeting. The (removed/blinded) meeting will be held at the (removed/blinded) in association with the (removed/blinded), from January 31, 2020 to February 3, 2020. Subjects recruited will volunteer to participate and will have specification of the risks and benefits of the study with completion of a consent form, free of coercion. Novice or trainee will be defined as resident orthopaedic surgical trainees in their senior (post-graduate year (PGY) 4 and 5) years. Expert will be defined as licenced practicing orthopaedic surgeons with shoulder fellowship training and current members of the (removed/blinded). Subject recruitment will be multi-institutional.

Once subjects are identified and consented, they will be randomized into training groups. The novice group will be blocked randomized based on year of study to attempt to produce consistent groups of surgical experience. The study will follow CONSORT recommendations and be presented in this format. The iVR intervention will consist of a reverse shoulder arthroplasty (RSA) for a superior wear pattern glenoid and the use of a metal augment to account for bone loss. The iVR intervention incorporates use of head mounted display (HMD) with sound and visual output, controllers with haptic (simulated vibration and resistance) feedback and position tracking will be used as is standard in contemporary iVR. The control intervention will be considered traditional learning method and will be in the form of a technical instructional video chosen by multiple study team members to best match the iVR intervention in terms of teaching key steps of RSA implantation. These key steps were decided on by multiple, expert shoulder surgeons and the authorship. The video used will be:

https://www.vumedi.com/video/reverse-shoulder-with-mini-humeral-tray-and-augmented-baseplate/

Both intervention and control groups will complete a demographic questionnaire that will collect baseline demographic information, experience with shoulder surgery and shoulder arthroplasty and familiarity with surgical simulation in education and that of iVR.

Both intervention groups will then complete their allocated training. Following completion of the training, a surprise written knowledge test will be provided for completion. The knowledge test will pertain to elements available in both training including types of glenoid wear patterns, ideal location of the guidepin for baseplate implantation, and amount of reaming. Both groups will additionally complete questionnaires on their perception of the quality of their respective training and a confidence scale.

Participants will be brought to the simulation center laboratory for dissection of fresh-frozen cadaveric specimens from scapula to hand. Consultant shoulder surgeons and authors will be blinded to training modality and present for participant evaluation. Evaluators will be provided with an information session prior to conducting the experiment to assure compliance and standardization of rating. The evaluators will be instructed to not aid the novice participant in their implantation and will be instructed to ask oral knowledge questions. Implantation will be timed and recorded. Evaluators will be tasked with completing scores for an objective structured assessment of technical skills (OSATS) score and global ratings scale (GRS). The evaluators will also be asked to comment on the orientation parameters of the final implanted glenoid baseplate. The participants will also be asked these parameters separately from the evaluators.

Following completion of the cadaveric dissection, participants will complete a final questionnaire pertaining to their respective intervention and it’s perceived realism, ability to teach the defined task of glenoid exposure, enjoyment of learning activity/intervention and perceptions of use in surgical education. They will also complete repeat CS questionnaires.

2.1 Sample size calculation

The primary outcome will be a comparison of OSATS values. The sample size will be based on a previous study in iVR validation that demonstrated significant difference in performance between eight subjects in each training arm (Lohre R, Bois A, Athwal GS, Goel DP, (CSES) TCS and ES. Improved Complex Skill Acquisition by Immersive Virtual Reality Training: A Randomized Controlled Trial. J Bone Jt Surg. 2020;102(6):e26.) To achieve 80% statistical power (beta =0.02) using a 2-sided test at alpha=0.05, for the primary outcome measure (OSATS) a minimum of six subjects are required for each group based on a conservative estimate of 25% difference between training groups. There is no expectation of subject loss as this is a single interventional study. Educational studies suffer from convenience sampling and resident participation in the course is expected to exceed our requirements of six individuals per group, and as such we will recruit as many as possible.

3. Aims and objectives

The aim of the study is to determine if iVR can produce improved OSATS scores in complex skill acquisition in orthopaedic surgical residents. This is representative of improved real-world performance, and transfer validity of skill of the iVR module and system. Secondary aims include characterizing the effects of iVR on verbal and written knowledge acquisition, training time differences, and transfer of training (ToT), transfer effectiveness (TER), and cost effectiveness (CER) ratios. The iVR module also provides users with a Precision Score. The Precision Score is a virtual metric of performance similar to real-world metrics such as OSATS or GRS. The Precision Score however accounts for final, virtual product orientation parameters based on best available evidence. The Precision Score for the RSA is provided by time of each key step, appropriate completion of each step, and the 3-dimensional performance quality of each step. The final score is a composite of these performance areas including quality of final product. The Precision Score is provided to the user and will be collected for comparison to

real-world performance. Table 1 illustrates primary and secondary outcome measures to be collected in the study.

4. Outcomes

This section will outline the primary and secondary objectives used to address the aims and objectives. Analyses will be presented separately in section 6. Table 1 demonstrates primary and secondary outcome measures.

Outcome	Measure	Description	Variable Type	Range	Calculation	Interpretation
Primary	OSATS	Composite check list of key and sequential steps in a surgical procedure	Ordinal	Each step 0 or 1. A "1" indicates completion of the step. Composite step score of 20 for this experiment	Addition of each step to determine sum.	A higher OSATS score indicates more appropriately completed key procedural steps, and therefore more understanding and technical ability to perform the procedure as a whole
Secondary	GRS	Performance assessment in domains of: (1) respect for tissue, (2) time and motion, (3)	Ordinal	Each domain consists of a Likert-scale of 1-5. A higher score indicates greater alignment	Addition of each step to determine sum.	Each domain can be assessed individually, or collectively. A higher score in each domain or collectively

		instrument handling, (4) knowledge of instruments, (5) flow of procedure, (6) knowledge of specific procedure, (7) overall performance, (8) quality of final product		with better performance in that domain. Composite scores can be produced of each domain together.		, respectively, indicates improved performance. When evaluated using this by an expert, higher scales indicate higher performance more in line with expert expectations.
Secondary	Transfer of training (ToT)	A comparison of skill achievement by those trained using the experimental simulation versus a control	Numerical – expressed as percent	A negative value indicates simulator training detracts from skill acquisition. A value of 0% indicates no effect of the simulator. A value of 100% indicates that an individual could reach proficienc	$ToT = \left(\frac{T_{video(cadaver)} - T_{iVR(cadaver)}}{T_{video(cadaver)}}\right)$ 100%	The percentage value indicates how much skill is gained using simulation training compared to the control group. A higher value indicates less dependency on real-world training and a

				y only using the simulator, not requiring any additional training.		reduction in learning curve of procedures.
Secondary	Transfer Effectiveness Ratio (TER)	A comparison of skill achievement incorporating task completion time	Numerical	Values from 0 to beyond 1.0. Values of 1.0 or greater indicate training is more effective in simulator than real life	$TER = \left(\frac{T_{video(cadaver)} - T_{iVR(cadaver)}}{T_{iVR(simulated)}}\right)$	Indicates how much real world training time is saved by using a simulator.
Secondary	Cost-Effectiveness Ratio (CER)	A cost saving comparison between simulator training and traditional training	Numerical	Values from 0 upwards. Higher values indicate greater cost savings	$CER = \frac{TER}{\left(\frac{Cost(iVR)}{Cost(control)}\right)}$	Indicates how much money is saved using a simulator compared to a control by incorporating training time and TER
Secondary	Confidence Scale (CS)	A developed psychometric scale adapted from (Grundy, S. E. (1993).	Ordinal	Likert-scale of 1-5, consisting of 6-questions. The higher the value, the	n/a	Higher cumulative scores for each Likert-type question corresponds to increasing

		The Confidence Scale. Nurse Educator, 18(1), 6–9.)		more confident the performance		confidence in performance
Secondary	Perception survey	Questions pertaining to: Enjoyment Realism Teaching capacity	Ordinal	Likert-scale questions from 1-5. Higher values indicate greater performance in each domain		Enjoyment: higher scores indicate greater enjoyment Realism: higher scores indicate greater realism and thus, face validity Teaching capacity: higher scores indicate greater ability to teach and thus, content validity
Secondary	Knowledge scores: Verbal Written	Questions pertaining to shoulder replacement principles and specific pathology identificati	Numerical	Cumulative scoring from 0 to 16 for written, and 0 to 6 for verbal	-	Higher scores indicate improved knowledge and recall.

		on				

Table 1. Primary and secondary outcome measures of the study

4.1 Primary outcome

The primary outcome will be translation of skill to an operative scenario measured by OSATS scores.

4.2 Secondary outcomes

Other aspects of validity including face (defined as realism) and content (ability of intervention to teach defined task) will be determined through questionnaire response. Other secondary outcomes include Precision Score values and correlation to OSATS, GRS scores, user confidence by CS questionnaires, overall implant orientation, ToT, TER, and CER.
Additionally, perceptions of enjoyment and perceived use in surgical education by the interventions will be measured by questionnaires.

5. Populations and subgroups to be analyzed

5.1 Populations

Per protocol (PP) analysis will be used as the primary population for the analysis, with analysis performed for all recruited subjects completing all of the outcomes and questionnaires appropriately and completely.

5.2 Subgroups

Subgroups will be analyzed based on the PP population.

Novice:
Defined novice/trainee group will be analyzed based on year of study for primary and secondary outcomes for both iVR and traditional learning (video) groups.

6. Analyses

Analyses will be performed through R platform for statistical computing. Data will be tested for normality and compared using descriptive statistics denoting mean and standard deviation (SD) for normal and mean and inter-quartile range (IQR) for non-normal data.

6.1 Primary outcomes

Analysis will be comparative of intervention groups (iVR versus video training) for OSATS scores. Values will be tested for normality and presented using descriptive statistics. Results will be considered significant at $p<0.05$. Data will be handled as a complete-case analysis.

6.2 Secondary outcomes

Descriptive statistics will be used to compare intervention groups (immersive VR versus traditional learning via video training). Student's t-test will be performed for direct comparison of means for normally distributed data for summative scores and Likert scales. Chi square testing will be performed for normally distributed single Likert-type data. Pearson product correlation will be used to determine similarity and correlation between ratings scales. Cronbach's alpha will be utilized to determine reliability of Likert scales. Results are to be considered significant at a $p<0.05$.

7. Missing data

PP population will be used. All data entries will be assured to be completed at time of study. Missing data will result in elimination of subject from the study and subsequent analyses.

***Title*:**
Effectiveness of Immersive Virtual Reality Training Compared to Instructional Technical Surgical Video: A Randomized Controlled Trial

Study Protocol
Version 2.0
Date January 15, 2020

Principal Investigator
Name: *Danny P. Goel*
Title: *Clinical Associate Professor*
Clinical Program: *UBC Orthopaedics*
Department: *Orthopedics*
CEO, Co-Founder: PrecisionOS Technology

Co-Investigator(s) *(if applicable)*
Name: *J Pollock*
Title: Clinical Associate Professor
Program: Surgery
Department: Orthopedics

Name: *Aaron Bois*
Title: Clinical Associate Professor
Program: Surgery
Department: Orthopedics

Name: *George Athwal*
Title: Clinical Associate Professor
Program: Surgery
Department: Orthopedics

Name: *Ryan Lohre*
Title: *Resident PGY 4*
Program*: UBC Orthopaedics*
Department*: Orthopaedics*

INTRODUCTION AND BACKGROUND

Medical education is changing secondary to expectation of flexible work-load, financial and time constraints.[1–4] The effect of this on surgical training potentially predisposes trainees to reduced operative exposure and experience.[5,6] A recent study highlights the majority of general surgery residents lacking confidence to begin independent surgical practice following graduation and that most are less likely to perform core procedures in the latter years of their training.[5] We have also noted a similar finding in orthopedic residents at a large Canadian teaching center on commonly observed fracture cases (Hunter et al unpublished data). Volume-outcome relationships have been previously demonstrated in orthopaedic surgery, as has time-action analysis for improved patient outcomes and efficiency, respectively.[7–16] Of particular interest is shoulder arthroplasty, demonstrating well-defined volume-outcome relationship on post-operative outcomes and associated cost given the technical complexity.[17–25] Reverse shoulder arthroplasty is a complex procedure requiring familiarity and confidence in surgical exposure, component insertion and positioning. Exposure of the glenoid and soft tissue management are crucial for stable prostheses and functional patient outcomes.[22–25] Surgical simulators have been produced to combat low volume technical tasks and to provide concrete reproducible experience, however the majority of these simulators currently lack fidelity, generalizability, as well as demonstrated validity and reliability despite consensus recommendations.[5,6,34–36,26–33]

Virtual Reality (VR), first coined in 1986 by Jaron Lanier, has expanded from the entertainment industry to clinical medicine in the preceding decades. This is based on its unique ability to replicate scenarios and environments while teaching skills in a potentially cost-effective manner. Simulator training is currently advocated by numerous surgical organizations, including the American Academy of Orthopaedic Surgeons (AAOS) however recent systematic reviews reveal the paucity of literature available for VR training in orthopaedic surgery.[37,38] Despite this, a recent study highlights the limited interaction of orthopaedic surgeons with VR or simulation experience, though the majority recognizing the role and benefit in practicing procedures and spatial orientation.[39] VR utilizes a combination of equipment including a three-dimensional (3D) rendering capable computer, head mounted display (HMD) and controllers with position trackers. Increasingly common is the addition of haptic feedback to VR to recreate sense of touch, vibration and motion.[38,40] In the field of orthopaedics, VR has demonstrated greatest potential in application for education secondary to the modification in the training environment.[28] VR systems provide continuous uninterrupted availability with available mentorship provided through immediate metrics, the ability for repetition, and outcome measures for task completion.

Training a single surgeon in the operating room has been estimated to cost roughly $48000 in the US. This is partly associated with increased operating room time with surgical trainees present, accounting for approximately 11 184 minutes of operating room time lost per trainee over four years.[30] Complications in improper surgical technique, reduction, or implant positioning in orthopaedic trauma surgery has additionally been previously demonstrated. The financial implications are also relevant where surgical inefficiency in Canada ranges from $621.60 to $2288.94 CAD per hour in a study incorporating multiple Canadian Hospitals.[10,41]

We have previously completed a randomized controlled trial of senior (PGY4 and PGY5) orthopaedic residents at the Canadian Shoulder and Elbow Society (CSES) 2019 resident training course. Residents from across Canada were randomized to training using an immersive VR simulator (PrecisionOS Technology, Vancouver, BC, Canada) versus a didactic technical journal article (representing traditional training methods) outlining steps to complex glenoid exposure. In this study, residents trained on VR demonstrated a 570% reduction in training time with equivalent knowledge scores on verbal and written testing, and 150% improvement in cadaveric dissection time with improved instrument handling scores measured by validated Objective Structured Assessment of Technical Skills (OSATS) metrics. This simulator was validated in all domains including transfer of skill and represented the first study of its kind in orthopaedic education (pending publication). This study laid the groundwork for subsequent examinations of transfer of learned skill. Resident education in orthopaedics utilizes multiple media sources for education. Technical surgical videos pertaining to specific implants are frequently read and used by residents and consultant orthopaedic surgeons. Surgical video online resources provide these videos to aid surgeons in developing knowledge of implant specific use. The use of these videos is

pervasive in programs across Canada and the world. We wish to further address the transfer of skill through VR training by comparing it to the well-established use of technical surgical videos in learning implant specific reverse shoulder arthroplasty. Additionally, the efficiency of learning using VR or traditional media systems should be evaluated using validated methods such as transfer of training (ToT) or transfer effectiveness ratios (TER).

PURPOSE AND JUSTIFICATION

Production of an immersive VR suite with haptic and user metric feedback would be an advance over current bench top simulator technology, allowing for greater immersion and interaction, leading to better understanding of surgical planning and implementation. The development of this technology could provide trainees with immersive levels of training not previously seen, with improved learning of technical skills over media such as manufacturer technical documents. The effectiveness of training and efficiency of training of the novel immersive VR training systems need to be evaluated as they are increasingly incorporated into competency based, contemporary residency education.

Research Question: Can immersive VR improve the efficiency and competency of technical skill acquisition in senior orthopaedic surgery residents over technical surgical instructional video in learning reverse shoulder arthroplasty?

Research Objective(s):

Primary: To determine if immersive VR is superior to technical surgical instructional video teaching in acquisition of technical skills in learning reverse shoulder arthroplasty

> Technical skill outcomes will be determined by Objective Structured Assessment of Technical Skills (OSATS), a Global Ratings Scale (GRS), and competency assessment (pass/fail) by fellowship trained subspecialty shoulder surgeons

Secondary: (1) To determine the efficiency of learning of immersive VR compared to manufacturer specific technical instructional document training of senior residents in learning reverse shoulder arthroplasty.

1. Efficiency will be determined by transfer of training (ToT) and transfer effectiveness ratio (TER)
2. To validate a created VR scoring system with real-world performance (this will include the use of CT scans of cadaveric specimens)

METHODS

Study Design:

A randomized, blinded intervention-control trial directly comparing immersive VR versus technical surgical instructional video training for teaching of reverse shoulder arthroplasty to senior orthopaedic surgery residents will be conducted.
Residents attending the Canadian Orthopaedic Association (COA), Canadian Shoulder and Elbow Society (CSES) resident training course in Ottawa will be consented for participation. Once consented, residents will be randomized to one of two groups using a computerized blocked protocol based on year of study (R4 and R5) to assure equal level of training between control (technical surgical instructional video) and intervention (immersive VR) groups. Every participant will complete a demographic questionnaire to determine age, handedness, visual correction (eyeglasses), familiarity with shoulder surgery, number of previous courses attended, and familiarity with simulation training and VR. Within the demographic questionnaire will be six questions regarding confidence of performing a reverse shoulder arthroplasty using a modified confidence scale (CS). The demographic questionnaire format will be of Likert-scale responses.
The control group will receive training on completion of a reverse shoulder arthroplasty using a technical surgical instructional video. The control group will be provided as much time as they require to watch the video, including repetition if desired during which they will be timed for completion.

The intervention (VR) group will receive training on completion of a reverse shoulder arthroplasty using an immersive VR simulator (PrecisionOS Technology, Vancouver, BC, Canada). The VR simulator utilizes an HMD producing 3D visuals with haptic controllers for an immersive operating room experience. The module produced consists of the key steps in performing a reverse shoulder arthroplasty using virtual versions of the equipment used in the real procedure. Prior to initiation, participants will be provided with a safety and training demonstration on the use of the VR module by study personnel. The intervention group will similarly be provided as much time as they desire with available repetition as they see fit. The VR group will be timed to task completion as the control group for comparison. The VR module will provide users with a score based on time to completion of key steps, and performance of key steps such as guide-pin insertion and overall glenoid baseplate orientation. These positioning scores will be based on clinically relevant cut-off values seen to affect implant longevity. These scores will be subsequently compared to previously validated GRS and OSATS scores for validation purposes.

Both groups will then be taken to a technical skills laboratory where they will be paired with an assessor (fellowship trained, consultant shoulder surgeon and member of CSES). The assessors will be blinded to the training received by the participant. The participants from both groups will then complete a reverse total shoulder arthroplasty using the same equipment used in either the control (technical surgical instructional video) or intervention (VR) learning activity while being assessed on fresh frozen cadavers (scapula to hand). The assessor will use an OSATS, GRS, and overall competency assessment for the resident during the procedure and for evaluation of the finished product. The assessor will also time the resident for time to task completion, which will be determined by the resident when they explicitly express that they are satisfied with final implantation. The participants in both groups and the blinded assessors will be asked to determine implant parameters once the reverse shoulder arthroplasty has been completed. Following this, participants will complete a survey assessing their enjoyment of learning activity, perceived benefit to continued use in learning orthopaedic skills, and a re-assessment of their confidence following training modality using the modified confidence scale (CS). The post-cadaveric questionnaire format will be of Likert-scale responses. The cadaveric specimens with inserted glenoid baseplates will be CT scanned to provide 2D and 3D reformats. This will allow for determination of the implant orientation parameters including version, inclination, rotation, and offset. These parameters will be compared to the VR scores to determine correlation between the VR training and real-world task completion.

Sampling Design and Subject Selection:

Subjects will be recruited from attendance at the Canadian Shoulder and Elbow Society (CSES) annual meeting by volunteering to participate. Subjects recruited will be in their PGY4 or PGY5 years of orthopaedic residency training and will be from multiple Canadian institutions. Once participants are recruited, study personnel will randomize groups to intervention (VR) or control (technical surgical instructional video) groups via a blocked randomization process in statistical computing software R (R Foundation for Statistical Computing, Vienna, Austria).

A recent systematic review comparing validity assessments of surgical orthopaedic simulators demonstrates a breadth of cohort variability, with 17.7% to upwards of 50% differences in reported outcome measures between novice/intermediate and expert groupings. TER ratios have seen variations from 7-42% in early VR simulators for novice surgeons. These studies predominantly focus on VR simulators lacking the immersion of contemporary VR, particularly the system from PrecisionOS. Similarly, the majority of VR simulator research in orthopaedics pertains to arthroscopic surgery which utilizes different global ratings scale outcome measures. The authors of this proposal have recently completed a similar randomized controlled trial at the 2019 CSES meeting (REB approval obtained from the University of Calgary) comparing senior resident VR training to traditional didactic scientific journal training. This resulted in a significant difference in cadaveric task completion time with n=8 in both groups. This study is presently pending publication and in review. As such, for power determination of comparison of VR trained to control group of novice surgeons utilizing a 2-sided test at 5% significance (alpha = 0.05) and to achieve 80% statistical power (beta = 0.02), considering a representative estimated difference of 25% (which we have seen in our previous study, and is conservative regarding similar literature) in combined outcome measures, six subjects will be required for each cohort.

Inclusion Criteria:

1. Individuals registered in licensed post-graduate orthopaedic residency programs attending the CSES resident course and consenting to participation.

Informed Consent:

There will be no direct patient interaction, therapeutic intervention, or other diagnostic or therapeutic intervention related to patient care. Participants will be provided with a consent form outlining the study. Information gathered on participants will not include direct identifiers aside from demographics of age, gender, and training experience. Questionnaires collected will be de-identified using study codes. The research demonstrates minimal risk to subjects involved as per TCPS2 Chapter 2, and Chapter 10, as the proposed study is observational, does not allow for direct identification of patients, is not staged, and is non-intrusive.

Study Procedures

Randomization: Subjects will be blocked randomized based on year of study (R4 or R5) using computer software.

Intervention: Study participants will voluntarily complete three questionnaires as well as an activity session utilizing either a technical surgical instructional video or VR to learn reverse shoulder arthroplasty followed by a cadaveric activity in the surgical skills laboratory.

Study Visits: Participants will voluntarily complete study requirements at a single visit during the CSES meeting of which they are electively attending.

Follow-up Visits: No follow-up visits of participating subjects.

Data Collection:

Data collection is prospective following randomization of study participants and blinding of expert raters. Data will be collected in a single setting. Voluntary participants will complete a pre-activity questionnaire identifying age, and gender as well as responses to a number of other questions regarding familiarity with shoulder arthroplasty and surgical simulation/VR as seen in appendix. Post-questionnaire data collected will include questions relating to realism and applicability in learning of the compared modalities and confidence. Questionnaires will be performed during the CSES course on paper/hard copy documents. PrecisionOS, the Lead Researcher, and Co-Researchers will have access to this de-identified data. Hardcopy questionnaires will be retained by the Lead Researcher and kept with PrecisionOS, in a locked and secure office. The hardcopy data will be retained for a period of 10 years. Only designated research personnel will have access to the key to participant de-identified study codes and this will be retained in their locked and secure office. Data will be tabulated to digital format, which will be collected, encrypted and stored on computers owned by the Lead Researcher. This data will then be stored on a secure server. Data will not be transferred out of Canada as per FIPPA. The study will take place once ethics approval is obtained and conclude at the end of the CSES course.

Measures:

Participant specific measures to be collected will include demographics, questionnaire responses, and task completion in an anonymous fashion.
Primary outcome of comparison of VR to technical surgical instructional video teaching will be assessed by OSATS, GRS, and competency grade provided by evaluators during the sawbone session. Secondary outcomes will be determined using the ToT and TER, overall time difference of task completion between groups, and CER. The cadaveric scapula will be assessed for implant version, inclination, rotation, and offset in the glenoid and recorded for each resident.
Data collection sheet including measures assessed can be seen in appendix.

Analytical Plan

Normality testing will be performed via Shapiro-Wilk test with subsequent mean comparative statistics to determine difference between VR and control groups. Descriptive statistics will be conducted. Likert questionnaire responses will be treated as Likert-scale and Likert-type data using descriptive statistics. Reliability testing for outcome scales in determining internal consistency will be assessed by Cronbach's alpha.

Ethical considerations

Potential Benefits: Study participants will receive expert instructional information regarding shoulder arthroplasty by participating. No remuneration for participation will occur. Longitudinal benefits could include improved technical skills in reverse shoulder arthroplasty.

Potential Risks: No direct risks to study participants involved. Subject recruitment is free of coercion, and only de-identified demographic data used with adherence to FIPPA and TCPS2 guidelines for data handling and storage.

Subject Safety Provisions: No direct risks to study participants. Occasionally use of immersive VR can produce feelings of nausea. This will be dictated to study participants prior to commencing the study, and participants are free to withdraw at any time. Safety provisions include data handling and transfer of patient information with adherence to FIPPA and TCPS2 guidelines and all attempts to store and utilize de-identified data.

Ethics Approval: Fraser Health Research Ethics Board (REB); Calgary REB

Plans for Publication and conference presentations:
Publication and conference presentations will be conducted following satisfaction of primary outcome and hypothesis testing. Specific editorial has not been determined, though likely conferences will include the Canadian Orthopaedic Association annual meeting, Orthopaedic Trauma Association annual meeting, American Academy of Orthopedic Surgeons and BC Orthopaedic Association Ortho update annual meeting.

References:

1. Wang H, Wang F, Newman S, et al. Application of an innovative computerized virtual planning system in acetabular fracture surgery : A feasibility study. 2016;47:1698-1701. doi:10.1016/j.injury.2016.05.006
2. Saadat L V, Dahlke AR, Rajaram R, et al. Program Director Perceptions of Surgical Resident Training and Patient Care under Flexible Duty Hour Requirements. *J Am Coll Surg*. 2016;222(6):1098-1105. doi:10.1016/j.jamcollsurg.2016.03.026
3. Bilimoria KY, Chung JW, Hedges L V, et al. National Cluster-Randomized Trial of Duty-Hour Flexibility in Surgical Training. *N Engl J Med*. 2016;374(8):713-727. doi:10.1056/NEJMoa1515724
4. Desai S V, Asch DA, Bellini LM, et al. Education Outcomes in a Duty-Hour Flexibility Trial in Internal Medicine. *N Engl J Med*. 2018;378(16):1494-1508. doi:10.1056/NEJMoa1800965
5. George BC, Bohnen JD, Williams RG, et al. Readiness of US General Surgery Residents for Independent Practice. *Ann Surg*. 2017;266(4):582-594. doi:10.1097/SLA.0000000000002414
6. Blay E, Hewitt DB, Chung JW, et al. Association between Flexible Duty Hour Policies and General Surgery Resident Examination Performance: A Flexibility in Duty hour Requirement for Surgical Trainees (FIRST) Trial Analysis. *J Am Coll Surg*. 2017;224(2):137-142. doi:10.1016/j.jamcollsurg.2016.10.042
7. Poeze M, Verbruggen JPAM, Brink PRG. The relationship between the outcome of operatively treated calcaneal fractures and institutional fracture load. A systematic review of the literature. *J*

Bone Joint Surg Am*. 2008;90(5):1013-1021. doi:10.2106/JBJS.G.00604

8. Joanne P J Minekus, R E, Rozing PM, et al. Factors infl uencing the surgical process during shoulder joint replacement : Time-action analysis of fi ve different prostheses and three different approaches. 2005;11(1):14-21.

9. Minekus JPJ, Rozing PM, Valstar ER, Dankelman J. Evaluation of humeral head replacements using time-action analysis. :152-157. doi:10.1067/mse.2003.14

10. Meeuwis MA, Jongh MAC, Roukema JA, Heijden FHWM, Verhofstad MHJ. Technical errors and complications in orthopaedic trauma surgery. *Arch Orthop Trauma Surg*. 2016;136(2):185-193. doi:10.1007/s00402-015-2377-5

11. Minekus JPJ, Rozing PM, Nelissen R, Dankelman J. Identifying error pathways during elbow and knee replacements. *Clin Orthop Relat Res*. 2005;(437):121-127.

12. Ward CM, Kuhl TL, Adams BD. Early complications of volar plating of distal radius fractures and their relationship to surgeon experience. *Hand (N Y)*. 2011;6(2):185-189. doi:10.1007/s11552-010-9313-5

13. Wierks C, Scd RLS, Ji JH, Mcfarland EG. Intraoperative and Early Postoperative Complications. 2009:225-234. doi:10.1007/s11999-008-0406-1

14. Rasuli KJ, Gofton W. Percutaneously assisted total hip (PATH) and Supercapsular percutaneously assisted total hip (SuperPATH) arthroplasty : learning curves and early outcomes. 2015;3(13):1-7. doi:10.3978/j.issn.2305-5839.2015.08.02

15. Laffosse J-M, Chiron P, Accadbled F, Molinier F, Tricoire J-L, Puget J. *Learning Curve for a Modified Watson-Jones Minimally Invasive Approach in Primary Total Hip Replacement: Analysis of Complications and Early Results versus the Standard-Incision Posterior Approach*. Vol 72.; 2007.

16. Schoenfeld AJ, Sturgeon DJ, Burns CB, Hunt TJ, Bono CM. Establishing benchmarks for the volume-outcome relationship for common lumbar spine surgical procedures. *Spine J*. 2018;18(1):22-28. doi:10.1016/j.spinee.2017.08.263

17. Jain N, Pietrobon R, Hocker S, Guller U, Shankar A, Higgins LD. The relationship between surgeon and hospital volume and outcomes for shoulder arthroplasty. *J Bone Joint Surg Am*. 2004;86-A(3):496-505.

18. Weinheimer KT, Smuin DM, Dhawan A. Patient Outcomes as a Function of Shoulder Surgeon Volume: A Systematic Review. *Arthroscopy*. 2017;33(7):1273-1281. doi:10.1016/j.arthro.2017.03.005

19. Walch G, Bacle G, Lädermann A, Nové-Josserand L, Smithers CJ. Do the indications, results, and complications of reverse shoulder arthroplasty change with surgeon's experience? *J Shoulder Elb Surg*. 2012;21(11):1470-1477. doi:10.1016/j.jse.2011.11.010

20. Groh GI, Groh GM. Complications rates, reoperation rates, and the learning curve in reverse shoulder arthroplasty. *J Shoulder Elb Surg*. 2014;23(3):388-394. doi:10.1016/j.jse.2013.06.002

21. Kempton LB, Ankerson E, Michael Wiater J. A complication-based learning curve from 200 reverse shoulder arthroplasties. *Clin Orthop Relat Res*. 2011;469(9):2496-2504. doi:10.1007/s11999-011-1811-4

22. Matache BA, Lapner P. Anatomic Shoulder Arthroplasty: Technical Considerations. *Open Orthop J*. 2017;11(Suppl-6, M4):1115-1125. doi:10.2174/1874325001711011115

23. a.M. H, T.R. N, P. B. Reverse shoulder arthroplasty indications, technique, and results. *Tech Shoulder Elb Surg*. 2005;6(3):135-149. doi:10.1097/01.bte.0000169730.36840.4b

24. Nové-Josserand L, Clavert P. Glenoid exposure in total shoulder arthroplasty. *Orthop Traumatol Surg Res*. 2018;104(1):S129-S135. doi:10.1016/j.otsr.2017.10.008

25. Nerot C, Ohl X. Primary shoulder reverse arthroplasty: Surgical technique. *Orthop Traumatol Surg Res*. 2014;100(1 S):S181-S190. doi:10.1016/j.otsr.2013.06.011

26. McDougall EM. Validation of surgical simulators. *J Endourol*. 2007;21(3):244-247. doi:10.1089/end.2007.9985

27. Carter FJ, Schijven MP, Aggarwal R, et al. Consensus guidelines for validation of virtual reality surgical simulators. *Simul Healthc*. 2006;1(3):171-179. doi:10.1097/01.SIH.0000244452.43542.47

28. Morgan M, Aydin A, Salih A, Robati S, Ahmed K. Current Status of Simulation-based Training Tools in Orthopedic Surgery: A Systematic Review. *J Surg Educ*. 2017;74(4):698-716. doi:10.1016/j.jsurg.2017.01.005

29. Van Nortwick SS, Lendvay TS, Jensen AR, Wright AS, Horvath KD, Kim S. Methodologies for

establishing validity in surgical simulation studies. *Surgery*. 2010;147(5):622-630. doi:10.1016/j.surg.2009.10.068
30. Thomas GW, Johns BD, Kho JY, Anderson DD. The validity and reliability of a hybrid reality simulator for wire navigation in orthopedic surgery. *IEEE Trans Human-Machine Syst*. 2015;45(1):119-125. doi:10.1109/THMS.2014.2339324
31. Froelich JM, Milbrandt JC, Novicoff WM, Saleh KJ, Allan DG. Surgical simulators and hip fractures: A role in residency training? *J Surg Educ*. 2011;68(4):298-302. doi:10.1016/j.jsurg.2011.02.011
32. Lin Y, Wang X, Wu F, Chen X, Wang C, Shen G. Development and validation of a surgical training simulator with haptic feedback for learning bone-sawing skill. *J Biomed Inform*. 2014;48:122-129. doi:10.1016/j.jbi.2013.12.010
33. Vankipuram M, Kahol K, McLaren A, Panchanathan S. A virtual reality simulator for orthopedic basic skills: A design and validation study. *J Biomed Inform*. 2010;43(5):661-668. doi:10.1016/j.jbi.2010.05.016
34. Vaughan N, Dubey VN, Wainwright TW, Middleton RG. A review of virtual reality based training simulators for orthopaedic surgery. *Med Eng Phys*. 2016;38(2):59-71. doi:10.1016/j.medengphy.2015.11.021
35. Hung AJ, Zehnder P, Patil MB, et al. Face, content and construct validity of a novel robotic surgery simulator. *J Urol*. 2011;186(3):1019-1024. doi:10.1016/j.juro.2011.04.064
36. Sugand K, Mawkin M, Gupte C. Validating Touch Surgery™: A cognitive task simulation and rehearsal app for intramedullary femoral nailing. *Injury*. 2015;46(11):2212-2216. doi:10.1016/j.injury.2015.05.013
37. Nizard R, Ph D. Orthopaedic Surgery. *Arthrosc J Arthrosc Relat Surg*. 2016;32(1):224-232. doi:10.1016/j.arthro.2015.07.023
38. Kim Y, Kim H, Kim YO. Virtual Reality and Augmented Reality in Plastic Surgery : A Review. 2017:179-187.
39. Blyth P, Anderson IA, Stott NS. Virtual reality simulators in orthopedic surgery: What do the surgeons think? *J Surg Res*. 2006;131(1):133-139. doi:10.1016/j.jss.2005.08.027
40. Kalun P, Wagner N, Yan J. Surgical simulation training in orthopedics : current insights. 2018:125-131.
41. Goldstein L, Ondrejicka D. A LITERATURE REVIEW AND MICRO COSTING APPROACH TO DETERMINE THE COST OF ONE HOUR OF OPERATING TIME IN. 2015;3(9):2014.
