# Supplementary material for: Effectiveness of Immersive Virtual Reality on Orthopedic Surgical Skills and Knowledge Acquisition Among Senior Surgical Residents: A Randomized Clinical Trial
Source: JAMA Netw Open. 2020 Dec 28;3(12):e2031217. doi: 10.1001/jamanetworkopen.2020.31217 (PMC7770558; doi:10.1001/jamanetworkopen.2020.31217)
Supplement: Supplement 2. — eAppendix 1. Global Ratings Scale Document eAppendix 2. OSATS Checklist Document eAppendix 3. Confidence Scale Document eAppendix 4. Demographic Survey eAppendix 5. Knowledge Test Document eAppendix 6. Postactivity Control Group Questionnaire eAppendix 7. Postactivity Experimental Group (IVR) Questionnaire [file jamanetwopen-e2031217-s002.pdf]

Supplemental Online Content

Lohre R, Bois AJ, Pollock JW, et al. Effectiveness of immersive virtual reality on orthopedic surgical skills and knowledge acquisition among senior surgical residents: a randomized clinical trial. *JAMA Netw Open*. 2020;3(12):e2031217. doi:10.1001/jamanetworkopen.2020.31217

eAppendix 1. Global Ratings Scale Document
eAppendix 2. OSATS Checklist Document
eAppendix 3. Confidence Scale Document
eAppendix 4. Demographic Survey
eAppendix 5. Knowledge Test Document
eAppendix 6. Postactivity Control Group Questionnaire
eAppendix 7. Postactivity Experimental Group (IVR) Questionnaire

This supplemental material has been provided by the authors to give readers additional information about their work.

© 2020 Lohre R et al. *JAMA Network Open.*

eAppendix 1. Global Ratings Scale Document

Respect for Tissue				
1	2	3	4	5
Overly aggressive force used; ie aggressively malleted implants, did not carefully ream		Careful handling of bone but occasionally caused inadvertent damage		Used appropriate skill in drilling and did not plunge, reamed appropriate depths, careful prosthesis seating
Time and Motion				
1	2	3	4	5
Many unnecessary movements; did not use time efficiently to finish task		Efficient time/motion but some unnecessary moves		All steps done succinctly without needless movements or hesitation
Instrument Handling				
1	2	3	4	5
Did not load instruments correctly or safely		Competent use of instruments but appeared stiff/awkard/inexperienced		Fluid movements with instruments; loaded instruments correctly and demonstrated control at all times
Knowledge of Instruments				
1	2	3	4	5
No understanding of correct sequence or correct use of guides, reamers etc		Knew names of most instruments and used appropriate instruments when required		Obviously familiar with instruments and properties: correctly used and chose implant guides, sized, reamed

© 2020 Lohre R et al. *JAMA Network Open.*

				and knew when to check for seating etc
Flow of Procedure				
1	2	3	4	5
Frequently stopped operating and seemed unsure of next move		Demonstrated some forward planning with reasonable progression of procedure		Clearly demonstrated correct sequence of procedure (guidepin insertion, glenoid reaming, augment guide placement, correct reaming, trialing of baseplace and insertion of baseplate and glenosphere)
Knowledge of Specific Procedure				
1	2	3	4	5
Deficient knowledge of reverse shoulder arthroplasty; required specific instruction at most steps and unsure of insertion technique of reverse glenoid implant		Knew all important steps of procedure, only missing minor details of reverse augmented baseplate insertion		Demonstrated familiarity with all steps of procedure
Overall Performance				
1	2	3	4	5
Very poor		Competent		Clearly superior
Quality of Final Product				
1	2	3	4	5
Very poor; unable to		Competent; appropriately sized glenoid component		Clearly superior; ideal positioning

© 2020 Lohre R et al. *JAMA Network Open.*

complete, incorrect orientation of baseplate or grossly incorrect size, intraoperative fracture		and adequate orientation with room for improvement		

eAppendix 2. OSATS Checklist Document

Please ask these questions at the beginning of assessment:

1. Have you ever used the Zimmer Biomet Comprehensive Reverse Total Shoulder Arthroplasty system with augmented baseplate?

○ Yes

○ No

2. If you have used the Zimmer Biomet Comprehensive Reverse Total Shoulder Arthroplasty system with augmented baseplate, how many cases do you estimate you have used this system?

○ 0

○ 1-10

○ 10-20

○ >20

3. Where is the wear pattern on the glenoid you are looking at? (please use terms such as anterior, posterior, superior, inferior, etc)

© 2020 Lohre R et al. *JAMA Network Open.*

4. What condition predisposes a glenoid to this wear pattern?

1. What is the wear pattern? Please circle one or more below that best fits what you are seeing.

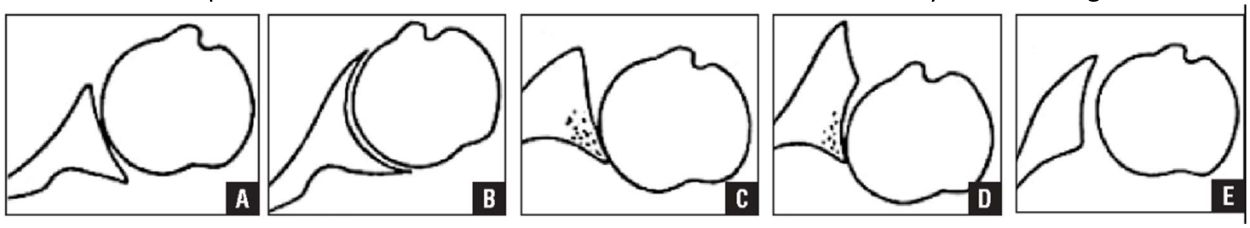

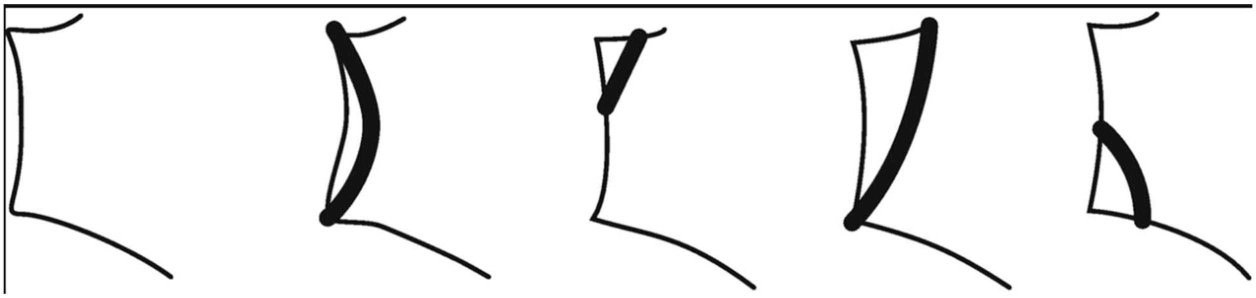

© 2020 Lohre R et al. *JAMA Network Open.*

Item	Not Done, Incorrect	Done, Correct
**Guidepin Insertion**		
1. Selects appropriate pin placement guide based on glenoid orientation (non-augmented, small, medium, large)	0	1
2. Selects appropriate “right” or “left” guide based on way the kickstand extends (not the operative side)	0	1
3. Centres the pin placement guide over the inferior glenoid in an appropriate position (if deformed, in location of best possible bone stock)	0	1
4. Able to insert Steinmann pin through guide in correct orientation (central axis of scapula with ~10 degrees inferior tilt)	0	1
5. Inserts the Steinmann pin to perforate the medial cortical wall to achieve stable (rigid) pin orientation	0	1
**Please ask this question after guidepin insertion:**<br>• What are acceptable locations of guidepin exit in the scapula?		
**Glenoid Reaming**		
1. Position the correct augment sizer (small, medium, large) over the Steinmann pin onto face of glenoid	0	1
2. Marks or verbally addresses/recognizes the need to account for the 50% line of the augment sizer	0	1
**Please ask this question regardless of performance for the above point**<br>• How deep do you want to ream this particular glenoid?		
3. Uses the appropriate Comprehensive Mini Baseplate Reamer	0	1
4. Is able to use the Comprehensive Mini Baseplate Reamer to ream the high-side and ream 50%	0	1

© 2020 Lohre R et al. *JAMA Network Open.*

5. Is able to assess the depth of ream using the augment sizer and if less than 50%, corrects it to 50%	0	1
**Choose augment**		
1. With the augment sizer over the Steinmann pin, is able to choose appropriate size (small, medium, large) augment for implantation	0	1
**Drill augment positioning hole**		
1. Places the 2.7mm peg drill guide appropriately on the glenoid (half-circle etch in location where augment is desired) ie at 50% line previously demarcated or area reamed to 50%	0	1
2. Drills the 2.7mm hole in correct location (opposite desired augment location) until drill is bottomed out and removes drill guide	0	1
**Place augment reamer guide and bushing**		
1. Places appropriately sized reamer guide over the Steinmann pin (reamer guide post placed into 2.7mm drill hole)	0	1
2. Removes Steinmann pin and replaces with 4.75mm guide screw by hand screw driver and assures it is fully seated	0	1
3. Inserts the reamer guide bushing onto the guide post, making sure it is seated	0	1
**Ream low-side of glenoid**		
1. Places appropriate sized reamer over bushing and reams until bottomed out	0	1
2. Removes reamer guide bushing, 4.75mm screw, and augment reamer guide	0	1
**Trial augment baseplate**		
1. Attempts a trial with the appropriate sized trial baseplate and orients it correctly	0	1
**Place augment baseplate**		
1. Correctly inserts the augmented baseplate (matches 2.7mm hole) and impacts it into correct position (ensures back is fully seated)	0	1
**Participant Total**		
**Max score**	20	

© 2020 Lohre R et al. *JAMA Network Open.*

eAppendix 3. Confidence Scale Document

Please answer the following six (6) questions with the response that best describes how you currently (**after** receiving your technical training module) feel about performing a reverse shoulder arthroplasty

Q1 I am certain that I could correctly perform a reverse shoulder arthroplasty

- Not certain at all
- Certain for only a few technical steps
- Fairly certain for a good number of technical steps
- Certain for almost all technical steps
- Absolutely certain of all technical steps

Q2 I feel that I can perform a reverse total shoulder arthroplasty without hesitation

- Not at all certain
- Certain for only a few technical steps
- Fairly certain for a good number of technical steps
- Certain for almost all technical steps
- Absolutely certain of all technical steps

Q3 My performance while performing a reverse total shoulder arthroplasty would convince a consultant shoulder surgeon that I was competent if viewed

- Not certain at all
- Certain for only a few technical steps
- Fairly certain for a good number of technical steps
- Certain for almost all technical steps
- Absolutely certain of all technical steps

Q4 I would feel sure of myself in performing a reverse total shoulder arthroplasty as primary surgeon

- Not certain at all
- Certain for only a few technical steps
- Fairly certain for a good number of technical steps
- Certain for almost all technical steps
- Absolutely certain of all technical steps

Q5 I would feel satisfied with my performance during a reverse shoulder arthroplasty as primary surgeon

© 2020 Lohre R et al. *JAMA Network Open.*

- Not certain at all
- Certain for only a few technical steps
- Fairly certain for a good number of technical steps
- Certain for almost all technical steps
- Absolutely certain of all technical steps

Q6 Overall, how confident are you to completely perform a reverse shoulder arthroplasty as primary surgeon?

- Very confident
- Confident
- Somewhat confident
- Somewhat not confident
- Not confident

eAppendix 4. Demographic Survey

Q1 What is your age?

____

Q2 What gender do you identify with?

- Male
- Female
- Prefer not to disclose

Q3 What is your current level of orthopaedic education?

- PGY 4
- PGY5

Q4 What is your dominant hand? (predominantly used to operate, write, or perform an important or challenging task)

- Left
- Right
- Use both equally

Q5 Do you have corrected vision? (glasses, contacts)

- Yes
- No

© 2020 Lohre R et al. *JAMA Network Open.*

Q6 How would you subjectively quantify your experience with shoulder surgical approaches?

- ◯ Not familiar
- ◯ Not very familiar
- ◯ Somewhat familiar
- ◯ Familiar
- ◯ Very familiar

Q7 How would you subjectively quantify your experience with reverse shoulder arthroplasty

- ◯ Not familiar
- ◯ Not very familiar
- ◯ Somewhat familiar
- ◯ Familiar
- ◯ Very familiar

Q8 How many shoulder surgery specific courses or lectures have you attended outside of your surgical training curriculum for which you are currently enrolled?

- ◯ 0-1
- ◯ 1-3
- ◯ >3

© 2020 Lohre R et al. *JAMA Network Open.*

Q9 How many reverse shoulder arthroplasty cases have you been primary surgeon (completed 100% of the case)?

- 0
- 1-10
- 10-20
- >20

Q10 Have you ever used the Zimmer Biomet Comprehensive Reverse Total Shoulder Arthroplasty system with augmented baseplate?

- Yes
- No

Q11 If you have used the Zimmer Biomet Comprehensive Reverse Total Shoulder Arthroplasty system with augmented baseplate, how many cases do you estimate you have used this system?

- 0
- 1-10
- 10-20
- >20

Q12 Have you utilized simulators in your surgical education, residency, or training courses?

- Yes
- No

© 2020 Lohre R et al. *JAMA Network Open.*

Q13 If you have utilized simulators in your surgical education, did you feel these improved your technical skill or knowledge?

- Not at all improved
- Somewhat improved
- Much improvement

Q14 Have you ever used any virtual reality products incorporating a head mounted display and position tracking controllers (including commercial gaming or entertainment)?

- Yes
- No

Q15 Have you ever used any virtual reality products in your surgical education, residency, or training courses?

- Yes
- No

Q16 If you have used virtual reality products in your surgical education, where did you use it and under what circumstances?

© 2020 Lohre R et al. *JAMA Network Open.*

Q17 If you have utilized virtual reality products in your surgical education, do you feel that these improved your technical skill or knowledge?

- Not at all improved
- Somewhat improved
- Much improvement

Q18 Have you ever used/watched instructional technical surgical videos in your orthopaedic training?

- Yes
- No

Q19 If you have used/watched instructional technical surgical videos in your surgical education, where did you use them and under what circumstances?

Q20 If you have used/watched instructional technical surgical videos in your surgical education, do you feel that these improved your technical skill or knowledge?

- Not at all improved
- Somewhat improved
- Much improvement

Q21 Have you ever used/watched a product specific instructional technical surgical video to learn a procedure, or to learn how to use an orthopaedic implant?

- Yes
- No

Q22 If you have used/watched a product specific instructional technical surgical video to learn a procedure, or to learn how to use an orthopaedic implant, did it improve your performance?

- Not at all improved
- Somewhat improvement
- Much improvement

Please answer the following six (6) questions with the response that best describes how you currently feel about performing a reverse shoulder arthroplasty

© 2020 Lohre R et al. *JAMA Network Open.*

Q23 I am certain that I could correctly perform a reverse shoulder arthroplasty

- Not certain at all
- Certain for only a few technical steps
- Fairly certain for a good number of technical steps
- Certain for almost all technical steps
- Absolutely certain of all technical steps

Q24 I feel that I can perform a reverse total shoulder arthroplasty without hesitation

- Not at all certain
- Certain for only a few technical steps
- Fairly certain for a good number of technical steps
- Certain for almost all technical steps
- Absolutely certain of all technical steps

Q25 My performance while performing a reverse total shoulder arthroplasty would convince a consultant shoulder surgeon that I was competent if viewed

- Not certain at all
- Certain for only a few technical steps
- Fairly certain for a good number of technical steps
- Certain for almost all technical steps
- Absolutely certain of all technical steps

Q26 I would feel sure of myself in performing a reverse total shoulder arthroplasty as primary surgeon

- Not certain at all
- Certain for only a few technical steps
- Fairly certain for a good number of technical steps
- Certain for almost all technical steps
- Absolutely certain of all technical steps

Q27 I would feel satisfied with my performance during a reverse shoulder arthroplasty as primary surgeon

- Not certain at all
- Certain for only a few technical steps
- Fairly certain for a good number of technical steps
- Certain for almost all technical steps
- Absolutely certain of all technical steps

© 2020 Lohre R et al. *JAMA Network Open.*

Q28 Overall, how confident are you to completely perform a reverse shoulder arthroplasty as primary surgeon?

- Very confident
- Confident
- Somewhat confident
- Somewhat not confident
- Not confident

© 2020 Lohre R et al. *JAMA Network Open.*

eAppendix 5. Knowledge Test Document

The following questions will act as an assessment of learning key steps in reverse shoulder arthroplasty using an augmented baseplate

Q1 What classification of glenoid morphology is commonly used to guide treatment?

____

Q2 What glenoid morphology(s) (based on the above classification) may benefit from the use of augments (bone or metal) during a reverse total shoulder arthroplasty?

____

Q3 Please list as many orientation parameters as you can for “ideal” placement of a glenosphere (ie. Version, etc)

____

____

____

____

____

Q4 Please list as many complications as you can for an incorrectly placed/oriented glenosphere

____

____

© 2020 Lohre R et al. *JAMA Network Open.*

____

____

____

Q6 Please list as many indications as you can for the use of a reverse shoulder arthroplasty?

____

____

____

____

____

© 2020 Lohre R et al. *JAMA Network Open.*

eAppendix 6. Postactivity Control Group Questionnaire

Please answer the following questions about your video learning activity in relation to your final cadaver examination.

Q1 Please enter your candidate code in the space provided

____

Q2 Did you enjoy the video learning activity?

○ Definitely yes

○ Mostly yes

○ Somewhat

○ Mostly not

○ Definitely not

Q3 Did you learn anything from watching the technical surgical instructional video?

○ Definitely yes

○ Mostly yes

○ Somewhat

○ Mostly not

○ Definitely not

© 2020 Lohre R et al. *JAMA Network Open.*

Q4 Did you feel that the technical surgical instructional video was easy to understand?

- Definitely yes
- Probably yes
- Somewhat
- Mostly not
- Definitely not

Q5 Did you feel that the technical surgical instructional video adequately prepared you for your subsequent cadaver reverse shoulder arthroplasty with augmented baseplate examination?

- Definitely yes
- Probably yes
- Somewhat
- Mostly not
- Definitely not

© 2020 Lohre R et al. *JAMA Network Open.*

Q6 What is your OVERALL impression of realism of the technical surgical instructional video?

- Very real
- Somewhat real
- Unsure
- Somewhat not real
- Very unrealistic

Q7 How proficient was the technical surgical instructional video in teaching relevant anatomy to reverse shoulder arthroplasty?

- Very good
- Somewhat good
- Neither good nor bad
- Somewhat not good
- Very poor

Q10 How proficient was the technical surgical instructional video in teaching key surgical steps in reverse shoulder arthroplasty?

- Very good
- Somewhat good
- Neither good nor bad
- Somewhat not good
- Very poor

© 2020 Lohre R et al. *JAMA Network Open.*

Q19 How proficient was the technical surgical instructional video in teaching key steps of insertion of the augmented baseplate in reverse shoulder arthroplasty?

◯ Very good

◯ Somewhat good

◯ Neither good nor bad

◯ Somewhat not good

◯ Very poor

Q12 How similar was the technical surgical instructional video to the cadaver reverse shoulder arthroplasty implantation in regard to teaching points and teaching surgical steps?

◯ Very similar

◯ Somewhat similar

◯ Unsure

◯ Somewhat not similar

◯ Very dissimilar

Q13 How well did the technical surgical instructional video prepare you for the cadaver reverse shoulder arthroplasty?

◯ Extremely well

◯ Very well

◯ Moderately well

◯ Slightly well

◯ Not well at all

© 2020 Lohre R et al. *JAMA Network Open.*

Q14 What is your OVERALL impression of the technical surgical instructional video in teaching key steps of augmented baseplate insertion for reverse shoulder arthroplasty?

- ○ Very good
- ○ Somewhat good
- ○ Neither good nor bad
- ○ Somewhat not good
- ○ Very poor

Q15 Would you use the technical surgical instructional video again to learn steps in reverse shoulder arthroplasty?

- ○ Yes
- ○ Maybe
- ○ No

Q16 Do you feel that repeated use of the technical surgical instructional video would provide additional benefit, or continued learning?

- ○ Definitely yes
- ○ Probably yes
- ○ Might or might not
- ○ Probably not
- ○ Definitely not

© 2020 Lohre R et al. *JAMA Network Open.*

Q17 Do you feel that a technical surgical instructional video has a role in surgical education?

- Yes
- Maybe
- No

Q18 Do you feel that the technical surgical instructional video would benefit surgical education for novice surgeons, such as residents or fellows?

- Yes
- Maybe
- No

Q19 Do you feel that technical surgical instructional video would provide benefit to expert surgeons performing shoulder arthroplasty?

- Yes
- Maybe
- No

Q20 Do you feel that Immersive Virtual Reality simulators have a role in surgical education in general?

- Yes
- Maybe
- No

© 2020 Lohre R et al. *JAMA Network Open.*

eAppendix 7. Postactivity Experimental Group (IVR) Questionnaire

Q1 Please enter your candidate code in the space provided

____

Q2 Did you enjoy your learning activity (VR module)?

- ○ Definitely yes
- ○ Mostly yes
- ○ Somewhat
- ○ Mostly not
- ○ Definitely not

Q3 Did you learn anything from your learning activity (VR module)?

- ○ Definitely yes
- ○ Mostly yes
- ○ Somewhat
- ○ Mostly not
- ○ Definitely not

© 2020 Lohre R et al. *JAMA Network Open.*

Q4 Did you feel that your learning activity (VR module) was easy to understand and to use?

- Definitely yes
- Probably yes
- Somewhat
- Mostly not
- Definitely not

Q5 Did you feel that your learning activity (VR module) adequately prepared you for your cadaver reverse shoulder arthroplasty experience?

- Definitely yes
- Probably yes
- Somewhat
- Mostly not
- Definitely not

© 2020 Lohre R et al. *JAMA Network Open.*

Q6 How would you rate the appearance of the anatomic structures in your learning activity (VR module) in regard to realism?

- ◯ Very real
- ◯ Somewhat real
- ◯ Unsure
- ◯ Somewhat not real
- ◯ Very unrealistic

Q7 How would you rate the appearance of the surgical equipment used in your learning activity (VR module) in regard to realism?

- ◯ Very real
- ◯ Somewhat real
- ◯ Unsure
- ◯ Somewhat not real
- ◯ Very unrealistic

© 2020 Lohre R et al. *JAMA Network Open.*

Q8 How would you rate the interactive ability of the anatomic structures in your learning activity (VR module) in regard to realism?

- ○ Very real
- ○ Somewhat real
- ○ Unsure
- ○ Somewhat not real
- ○ Very unrealistic

Q9 How would you rate the interactive ability of the surgical instrumentation with the anatomic structures during your learning activity (VR module) in regard to realism?

- ○ Very real
- ○ Somewhat real
- ○ Unsure
- ○ Somewhat not real
- ○ Very unrealistic

© 2020 Lohre R et al. *JAMA Network Open.*

Q10 How would you rate the user control scheme in regard to surgical operating room realism during your learning activity (VR module)?

- Very real
- Somewhat real
- Unsure
- Somewhat not real
- Very unrealistic

Q11 How would you rate the realism of the haptic feedback provided by the controllers when interacting with the anatomic structures during the VR module?

- Very real
- Somewhat real
- Unsure
- Somewhat not real
- Very unrealistic

© 2020 Lohre R et al. *JAMA Network Open.*

Q12 How ergonomic/comfortable was the control system used during your learning activity VR module)?

- Very ergonomic
- Somewhat ergonomic
- Unsure
- Somewhat not ergonomic
- Very not ergonomic

Q13 How would you rate the realism of the movement of the instruments during the VR module?

- Very real
- Somewhat real
- Unsure
- Somewhat not real
- Very unrealistic

Q14 What is your OVERALL impression of realism of the VR module?

- Very real
- Somewhat real
- Unsure
- Somewhat not real
- Very unrealistic

© 2020 Lohre R et al. *JAMA Network Open.*

Q15 How proficient was the VR module in teaching relevant anatomy to reverse shoulder arthroplasty?

- Very good
- Somewhat good
- Neither good nor bad
- Somewhat not good
- Very poor

Q18 How proficient was the VR module in teaching key surgical steps in reverse shoulder arthroplasty?

- Very good
- Somewhat good
- Neither good nor bad
- Somewhat not good
- Very poor

© 2020 Lohre R et al. *JAMA Network Open.*

Q19 How proficient was the VR module in teaching key technical steps of insertion of the augmented baseplate in reverse shoulder arthroplasty?

- ○ Very good
- ○ Somewhat good
- ○ Neither good nor bad
- ○ Somewhat not good
- ○ Very poor

Q20 How similar was the VR module to the cadaver reverse shoulder arthroplasty implantation in regard to teaching points and teaching surgical steps?

- ○ Very similar
- ○ Somewhat similar
- ○ Unsure
- ○ Somewhat not similar
- ○ Very dissimilar

Q21 How well did the VR module prepare you for the cadaver reverse shoulder arthroplasty?

- ◯ Extremely well
- ◯ Very well
- ◯ Moderately well
- ◯ Slightly well
- ◯ Not well at all

Q22 What is your OVERALL impression of the VR module activity in teaching key steps of augmented baseplate insertion for reverse shoulder arthroplasty?

- ◯ Very good
- ◯ Somewhat good
- ◯ Neither good nor bad
- ◯ Somewhat not good
- ◯ Very poor

Q23 Would you use the VR module again to learn steps in reverse shoulder arthroplasty?

- ◯ Yes
- ◯ Maybe
- ◯ No

© 2020 Lohre R et al. *JAMA Network Open.*

Q24 Do you feel that repeated use of the VR module would provide additional benefit, or continued learning?

- ○ Definitely yes
- ○ Probably yes
- ○ Might or might not
- ○ Probably not
- ○ Definitely not

Q25 Do you feel that the VR module has a role in orthopaedic surgical education?

- ○ Yes
- ○ Maybe
- ○ No

Q26 Do you feel that the VR module would benefit surgical education for novice surgeons, such as residents or fellows?

- ○ Yes
- ○ Maybe
- ○ No

© 2020 Lohre R et al. *JAMA Network Open.*

Q27 Do you feel that the VR module would provide benefit to expert surgeons performing reverse shoulder arthroplasty?

- ◯ Yes
- ◯ Maybe
- ◯ No

Q28 Do you feel that Immersive Virtual Reality simulators have a role in surgical education in general?

- ◯ Yes
- ◯ Maybe
- ◯ No

© 2020 Lohre R et al. *JAMA Network Open.*
