# Supplementary material for: Effectiveness of Immersive Virtual Reality on Orthopedic Surgical Skills and Knowledge Acquisition Among Senior Surgical Residents: A Randomized Clinical Trial
Source: JAMA Netw Open. 2020 Dec 28;3(12):e2031217. doi: 10.1001/jamanetworkopen.2020.31217 (PMC7770558; doi:10.1001/jamanetworkopen.2020.31217)
Supplement: Supplement 3. — Data Sharing Statement [file jamanetwopen-e2031217-s003.pdf]

Data Sharing Statement

Lohre. Effectiveness of Immersive Virtual Reality on Orthopedic Surgical Skills and Knowledge Acquisition Among Senior Surgical Residents. *JAMA Netw Open*. Published December 28, 2020.
doi:10.1001/jamanetworkopen.2020.31217

Data
Data available: No
